# The Role and Clinical Correlates of Complex Post-traumatic Stress Disorder in People With Psychosis

**DOI:** 10.3389/fpsyg.2022.791996

**Published:** 2022-03-16

**Authors:** Peter Panayi, Katherine Berry, William Sellwood, Carolina Campodonico, Richard P. Bentall, Filippo Varese

**Affiliations:** ^1^Division of Psychology and Mental Health, Manchester Academic Health Sciences Centre, University of Manchester, Manchester, United Kingdom; ^2^Complex Trauma and Resilience Research Unit, Greater Manchester Mental Health NHS Foundation Trust, Manchester, United Kingdom; ^3^Faculty of Health & Medicine, Division of Health Research, University of Lancaster, Lancaster, United Kingdom; ^4^School of Psychology and Computer Science, University of Central Lancashire, Lancashire, United Kingdom; ^5^Department of Psychology, University of Sheffield, Sheffield, United Kingdom

**Keywords:** trauma, psychosis, PTSD, CPTSD, mediation, disturbance of self-organisation

## Abstract

Traumatic experiences and post-traumatic stress are highly prevalent in people with psychosis, increasing symptom burden, decreasing quality of life and moderating treatment response. A range of post-traumatic sequelae have been found to mediate the relationship between trauma and psychotic experiences, including the “traditional” symptoms of post-traumatic stress disorder (PTSD). The International Classification of Diseases-11th Edition recognizes a more complex post-traumatic presentation, complex PTSD (cPTSD), which captures both the characteristic symptoms of PTSD alongside more pervasive post-traumatic sequelae known as ‘disturbances in self-organization’ (DSOs). The prevalence and impact of cPTSD and DSOs in psychosis remains to be explored. In the first study of this kind, 144 participants with psychosis recruited from North West United Kingdom mental health services completed measures assessing trauma, PTSD and cPTSD symptoms and symptoms of psychosis. Forty-percent of the sample met criteria for cPTSD, compared to 10% who met diagnostic criteria for PTSD. PTSD and DSOs mediated the relationship between trauma and positive symptoms, controlling for dataset membership. Both PTSD and DSOs mediated the relationship between trauma and affective symptoms but did not explain a significant proportion of variance in negative symptoms. Cognitive and excitative symptoms of psychosis did not correlate with trauma, PTSD or DSO scores. These findings indicate the possible value of adjunct therapies to manage cPTSD symptoms in people with psychosis, pending replication in larger epidemiological samples and longitudinal studies.

## Introduction

Traumatic life events and adverse childhood experiences may lead to various psychosocial difficulties. Perhaps the most notable consequence of such experiences is Post-Traumatic Stress Disorder (PTSD), characterized by re-experiencing (e.g., intrusive trauma memories), hyperarousal (e.g., irritability and hypervigilance) and avoidance of trauma reminders (World Health Organisation, 2019). Cohort studies have recorded a prevalence of PTSD as high as 7.8% in England (Lewis et al., 2019). PTSD has been shown to predict adverse physical and mental health outcomes (Gradus, 2017; Ryder et al., 2018), as well as reduced quality of life and social functioning (Balayan et al., 2014; Pagotto et al., 2015).

Following the classification of PTSD in the third edition of the Diagnostic and Statistical Manual of Mental Disorders (American Psychiatric Association, 1980), neither clinicians nor trauma survivors felt it captured the complex difficulties endorsed by victims of multiple or prolonged traumas. Hence, Herman (1992) introduced the concept of complex PTSD (cPTSD). This includes the above core symptoms of PTSD, as well as broader and more severe symptomatology (e.g., cognitive, affective, and relational disturbance). Following a body of empirical research demonstrating a quantitative (Hyland et al., 2017) and qualitative (Stadtmann et al., 2018) distinction between PTSD and cPTSD, the most recent International Classification of Diseases (11^th^ Edition; ICD-11, World Health Organisation, 2019) has recognized cPTSD as a separate, sibling diagnosis to PTSD (Karatzias et al., 2017). This includes the symptoms of PTSD as above, alongside other symptoms collectively referred to as ‘disturbances of self-organization’ (DSOs), including negative self-concept, emotional dysregulation and interpersonal difficulties. Prior studies indicate that core PTSD symptoms may be more severe in people with cPTSD (Wolf et al., 2015; Murphy et al., 2016), that cPTSD is associated with increased burden (Cloitre et al., 2020), and requires disparate treatment approaches to PTSD (UK Psychological Trauma Society, 2017).

Complex trauma histories are highly common among people with schizophrenia spectrum conditions (Trauelsen et al., 2015), with meta-analyses consistently implicating trauma as an aetiological factor in psychosis (Varese et al., 2012; Kelleher et al., 2013; Bell et al., 2019). Trauma exposure may lead to symptoms of PTSD – namely, re-experiencing and memory intrusions – that may be appraised as anomalous experiences (e.g., hearing insults or phrases of past abusers appraised as an external voice in the present moment) (Morrison et al., 2003). Systematic reviews have indicated that PTSD symptoms mediate the relationship between trauma and psychosis (Williams et al., 2018; Alameda et al., 2020; Sideli et al., 2020). Despite consistent evidence of the involvement of PTSD symptoms in the pathway between trauma and psychosis, there are no studies to our knowledge investigating the potential role of cPTSD.

The involvement of these additional post-traumatic sequelae in the trauma-psychosis relationship is plausible, considering previous studies which considered similar mediators. Systematic reviews have shown that emotion dysregulation, negative thoughts about the self and interpersonal difficulties mediate the relationship between trauma and psychosis (Williams et al., 2018; Alameda et al., 2020; Sideli et al., 2020). Meta-analytic evidence is concordant with these findings, showing that emotion dysregulation, negative self-concept and attachment difficulties predict specific psychotic symptoms following trauma exposure (Bloomfield et al., 2021). Further plausibility for the role of cPTSD in the pathway from trauma to psychosis stems from repeated childhood trauma, an risk factor for cPTSD (Cloitre et al., 2013) that is highly common among those with psychosis (Trauelsen et al., 2015). Similarly, PTSD is highly comorbid with psychosis (Buckley et al., 2009; Achim et al., 2011), moderating treatment outcomes and reducing quality of life (Grubaugh et al., 2011; Hassan and De Luca, 2015). This requires replication in a psychosis sample to inform assessment and intervention.

Negative symptoms of psychosis remain largely unexplained by PTSD. Strauss et al. (2011) found those meeting criteria for deficit schizophrenia (i.e., negative symptoms related to the illness itself lasting longer than 12 months; Carpenter et al., 1988) were at lower risk of PTSD than those displaying secondary negative symptoms. Consistently, subsequent meta-analytic evidence (Brand et al., 2018) has found small, non-significant effects of trauma-focused cognitive-behavioral therapies on negative symptoms. Together, these findings suggest PTSD symptoms may not mediate the relationship between trauma and negative psychotic symptoms. DSOs may, however, play a role in this relationship as opposed to PTSD, owing to their apparent clinical similarity with certain negative symptoms. For instance, DSOs may present as emotional numbing and anhedonia (Cloitre et al., 2013; Favrod et al., 2019) as well as social withdrawal (Griffiths and McLeod, 2020), consistent with negative symptom presentations in psychosis. The limited evidence-based interventions for negative symptoms and their associated burden (Correll and Schooler, 2020) makes this mediation hypothesis worth exploring.

Psychotic symptomatology is not restricted to positive and negative domains. Affective difficulties are also common in people with psychosis, with anxiety and major depressive disorders affecting up to 1 in 3 people at their first episode (Wilson et al., 2020). Systematic reviews suggest that these difficulties correlate with psychotic symptom severity, distress and content (Hartley et al., 2013), and decrease quality of life (Nevarez-Flores et al., 2019), making affective problems key targets in psychological interventions for psychosis. PTSD is associated with a greater risk of anxiety and depression (Spinhoven et al., 2014), and cPTSD even more so (Karatzias et al., 2019). Hence, post-traumatic sequelae may play a maintaining role in affective difficulties among people with psychosis.

Other symptom domains of psychosis have also been identified; namely, cognitive and excitative difficulties. PTSD symptoms do not correlate with cognitive difficulties in people with psychosis (Lysaker and LaRocco, 2008; DeTore et al., 2021). However, those with an alleged neurodevelopmental predisposition to psychosis may be at greater risk of childhood victimization, especially bullying from peers (Liu et al., 2021). Thus, this pathway may interact with a trauma pathway to psychosis. The positive/negative symptom solutions typically used in the scoring of the PANSS may not capture such cognitive difficulties; factor analytic studies have identified a more complex underlying structure to the PANSS comprising positive, negative, cognitive, affective and excitative symptoms (Wallwork et al., 2012; Shafer and Dazzi, 2019; Lim et al., 2021). Therefore, it is possible that post-traumatic sequelae more complex than PTSD – i.e., cPTSD – may lead to nuanced psychotic symptoms, such as cognitive/excitative symptoms. Given that adults with PTSD following childhood maltreatment scored significantly higher on cognitive dysfunction than those without such experiences (Nakayama et al., 2020), and also given that cPTSD is often associated with prolonged, repeated traumatic experiences during childhood, it is plausible that cPTSD may incur greater cognitive consequences than PTSD. Preliminary findings support this hypothesis, demonstrating that childhood trauma is linked to subjective and objective cognitive difficulties among people with psychosis, including working memory and attention (Reinhard et al., 2010; Vargas et al., 2019). Therefore, it is possible that DSOs rather than core PTSD symptoms may contribute to explaining cognitive and/or excitative symptoms of psychosis.

**FIGURE 1:**
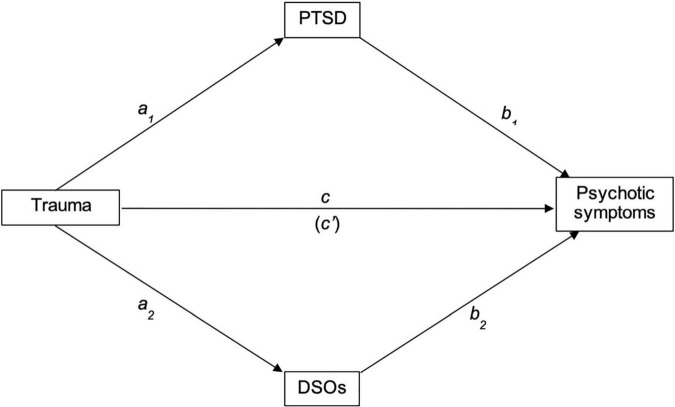
Proposed path of the relationships between trauma, PTSD, DSOs and psychotic symptoms. PTSD, Post-traumatic stress disorder; DSOs, Disturbances of self-organization.

This study aimed to describe rates of cPTSD and PTSD in a trauma-exposed sample of people with psychosis, assess clinical differences in symptom severity between trauma groups, and explore the relative contribution of cPTSD via PTSD and DSOs in explaining the relationship between trauma and psychotic symptoms. Using a parallel mediation framework (illustrated in Figure 1), we tested separate mediational models to test whether PTSD and DSO symptoms mediated between trauma and positive, negative, affective, cognitive and excitative symptoms.

## Materials and Methods

### Study Design

This study employed a correlational design, combining data collected from a feasibility randomized controlled trial testing the feasibility and acceptability of an Eye-Movement Desensitization and Reprocessing for psychosis intervention (the EASE trial) (Varese et al., 2020), and a previous research project within the University of Manchester Complex Trauma & Resilience Research Unit (Campodonico et al., 2021). Both samples were recruited from North West of England mental health services with ethical approval received from an National Health Service (NHS) research ethics committee.

### Participants

This study employed the baseline sample of the EASE trial (*n* = 66) and the full sample recruited by Capodonico and colleagues (*n* = 85), thus *N* = 151.

The inclusion criteria for the parent studies are reported in full elsewhere (Varese et al., 2020; Campodonico et al., 2021). Generally, these included adults with a schizophrenia-spectrum diagnosis (or who met diagnostic criteria), that were registered with local NHS mental health services, and had capacity to provide informed consent at the time of recruitment. Exclusion criteria for both studies were requirement of an interpreter, a primary diagnosis of substance misuse, intellectual disability or gross cognitive dysfunction.

In addition to those set out by the parent studies, inclusion criteria for this study included endorsement of at least one traumatic life event on the Trauma and Life Events checklist (TALE; Carr et al., 2018), with International Trauma Questionnaire (Cloitre et al., 2018) scores anchored to traumas identified by the TALE. Participants must also have completed the Positive and Negative Syndrome Scale (Kay et al., 1987).

### Measures

The Positive and Negative Syndrome Scale (PANSS; Kay et al., 1987) is a 30-item semi-structured clinical interview used to measure psychotic symptoms and general psychopathology. Items are scored on a Likert scale from 1 (“Absent”) to 7 (“Extreme”), with higher scores indicating more severe symptoms. This study employed a pentagonal model of the PANSS, in accordance with factor analytic evidence (Shafer and Dazzi, 2019; Lim et al., 2021). This is comprised of 5 factors: positive, negative, cognitive (measuring cognitive disorganization), affective (measuring anxiety and depression) and excitative (measuring activity and hostility); see Supplementary Table 1. Cronbach’s α in this sample for these subscales were good, ranging from .73 to .83, aside from the negative subscale, where α = 0.66. This is comparable to prior research (Peralta and Cuesta, 1994). All PANSS interviews were administered by trained and supervised research assistants/workers who completed thorough reliability assessments against “gold standard” scores produced by expert PANSS raters. Raters across datasets demonstrated excellent inter-rater reliability with ‘gold standard’ scores, with baseline intra-class correlation coefficients (ICCs) ranging from 0.86 to 0.94 in the EASE dataset, and 0.90 in the Campodonico dataset.

The Trauma and Life Events Questionnaire (TALE; Carr et al., 2018) is a 22-item self-report checklist assessing traumatic and difficult life experiences. Each event is scored for its occurrence, whether this was more than once, and whether this occurred when the participant was under/over 16 or both. The number of traumas endorsed is summed to derive a traumatic experiences score. The TALE demonstrates good test-retest reliability and convergent validity with related trauma measures (Favrod et al., 2019).

The International Trauma Questionnaire (ITQ; Cloitre et al., 2018) is an 18-item self-report scale assessing the presence and severity of PTSD and DSOs within the past month. Items are administered in relation to an index trauma identified on the TALE as affecting the individual most in the past month. PTSD and DSO subscales are each comprised of three symptom clusters, themselves comprised of two items each. Both subscales include three additional items measuring functional impairment associated with the symptoms captured by each subscale. All items are scored on a 5-point Likert scale from 0 (“Not at all”) to 4 (“Extremely”). As per the ITQ diagnostic algorithm, a probable diagnosis of PTSD is identified when a participant presents with a score of 2 or more on at least one item in each PTSD cluster, plus a score of 2 or more on at least one functional impairment item associated with these symptoms. The cPTSD threshold includes that of PTSD, as well as a score of 2 or more on at least one item in each DSO cluster, plus a score of 2 or more on at least one functional impairment item associated with these symptoms. PTSD and DSO items were totaled to derive continuous severity scores, with higher scores indicating higher severity. Scores within each DSO cluster were summed to derive continuous DSO scores for use in planned secondary analyses. Both PTSD and DSO subscales demonstrated high internal consistency in this sample (α = 0.83 and 0.87, respectively).

### Procedures

Participants were first introduced to either parent study by members of their clinical teams, and signposted to the research team, if interested in participating. Participants then met with a researcher for more information about the relevant study at a mutually convenient location or a digital meeting (during the COVID-19 pandemic in affected periods of the EASE trial). Information sheets were then provided, and informed consent taken. Following informed consent, participants completed a battery of measures that included the PANSS, TALE and ITQ, in addition to other measures (see Varese et al., 2020; Campodonico et al., 2021).

### Data Analysis

Diagnostic algorithms outlined above were applied to ITQ scores to group participants into those who met criteria for PTSD, cPTSD or neither. Independent samples t-tests were then used to explore mean differences in psychotic symptoms scores between these trauma groups. Correlation, regression and finally mediation analyses were used to examine the role of PTSD and DSO symptoms in the relationship between trauma and symptom dimensions. The Baron and Kenny (1986) requirement for the presence of a significant direct effect was used here as a conservative option to indicate subsequent mediation analyses, to minimize Type I error.

Out of 151 participants available from the parent studies, 144 people met inclusion criteria for this study. No cases were identified as multivariate outliers per Mahalanobis (1936), Cook’s (Cook, 1977) or Leverage analyses of distance. The data were then checked for normality and homoscedasticity; both assumptions were met per Curran and colleagues’ criteria (Curran et al., 1996).

Within this dataset, less than 20% of the data were missing. Little’s MCAR test was not significant, χ2 = 257.59(251), *p* = 0.37, suggesting the data were missing at random. Thus, the missing scores were imputed where possible. Eight participants were missing PANSS scores on specific items likely obfuscated by remote assessments in the EASE trial; these participants were excluded from analyses involving these items. Where one of the two scores on an ITQ symptom was missing, this was imputed with the score on the other item. Where an ITQ functional impact score was missing, this was imputed with the mean of the other two available scores. One participant chose not to respond to one item on the TALE; this was not imputed, as trauma checklists are likely not missing at random. Then, specific items were summed to derive continuous TALE, PTSD, DSO, and PANSS subscale scores.

Twenty-four participants in the EASE trial completed their assessments remotely due to the COVID-19 lockdown restrictions on face-to-face working. This impacted the confidence of negative PANSS ratings reliant on factors more easily observed in person (e.g., gesticulation). Analyses were rerun with these participants excluded (*n* = 120), to check the validity of the full dataset. This did not affect the outcomes of the study. As such, the following findings are in relation to the full combined sample.

To capture potential confounding differences between parent datasets, dataset membership was entered as a covariate in our analyses. This did not affect the outcomes of PANSS-negative or -affective analyses. As such, the analyses of these outcomes reported below do not include covariates. Dataset did, however, change the outcomes of analyses of PANSS-positive scores. Thus, the analyses thereof include dataset as a covariate. Bonferroni-corrected independent samples *t*-tests and bivariate Pearson correlations were used to assess the role of gender and age, respectively, as potential covariates. The only significant finding was a small, positive correlation between PANSS-cognitive scores and age (*r* = 0.19, *p* = 0.027). Age would therefore be included in analyses of PANSS-cognitive scores, but these were not indicated by bivariate correlations (see below).

## Results

### Descriptive Statistics

Demographic and clinical characteristics were aggregated across datasets, reported in Table 1. Participants reported a mean of 9.7 traumatic life experiences on the TALE. The most common experiences endorsed were loss or permanent separation from a close friend or relative (81%), bullying (70%), emotional (66%) and physical (62%) abuse.

**TABLE 1:** Bivariate Pearson correlations between PANSS subscales and trauma variables.

	Positive	Negative	Cognitive	Affective	Excitative	TALE score	PTSD	DSO	*M*	*SD*
Positive	–	0.13	0.21*	0.46**	0.12	0.26**	0.45**	0.38**	15.4	4.8
Negative		–	0.17	0.39**	–0.03	0.06	0.28**	0.28**	13.8	4.9
Cognitive			–	0.12	0.52**	–0.09	−0.17*	–0.15	13.1	4.3
Affective				–	–0.05	0.32**	0.55**	0.61**	14.5	5.2
Excitative					–	–0.13	–0.13	–0.10	4.9	2.3
TALE score						–	0.38**	0.37**	9.7	3.6
PTSD							–	0.59**	13.2	6.9
DSO								–	14.5	6.8

PANSS, Positive and negative syndrome scale; TALE, Trauma and life events checklist; PTSD, Post-traumatic stress disorder; DSO, Disturbances of self-organisation; M, mean; SD, standard deviation. *Significant at the p < 0.05 level; **Significant at the p < 0.01 level.

### The Frequency of Complex Post-traumatic Stress Disorder

The ITQ diagnostic algorithm was applied to ITQ scores of the sample to delineate groups of participants meeting ICD-11 criteria for PTSD and cPTSD. Among those who met criteria for a post-traumatic stress diagnosis (50.7%), cPTSD was far more common (40.3%) than PTSD (10.4%).

### Symptom Severity Between Trauma Group

A between-subjects MANOVA found a significant overall trauma diagnosis group differences across PANSS subscales (*F*_10,252_ = 4.702, *p* < 0.001, Wilk’s Λ = 0.710). Follow-up univariate ANOVAs detected significant differences on positive (*F*_2_ = 6.02, *p* = 0.003, η_p_^2^ = 0.09), negative (*F*_2_ = 5.94, *p* = 0.003, η_p_^2^ = 0.08) and affective (*F*_2_ = 17.16, *p* < 0.001, η_p_^2^ = 0.21) PANSS subscales. No significant differences among cognitive (*F*_2_ = 1.30, *p* = 0.276, η_p_^2^ = 0.02) or excitative (*F*_2_ = 6.14, *p* = 0.345, η_p_^2^ = 0.02) subscales were observed. *Post-hoc* Tukey’s tests were used to investigate significant differences between groups. These suggested that positive symptoms were significantly higher among those meeting criteria for cPTSD (*M* = 16.86, *SD* = 3.93) than those who met criteria for neither cPTSD nor PTSD (*M* = 13.93, *SD* = 5.13), as were negative symptoms (cPTSD: *M* = 15.41, *SD* = 5.40; none: *M* = 12.66, *SD* = 4.21). Affective symptoms were significantly higher among those meeting criteria for cPTSD (*M* = 17.51, *SD* = 4.22) compared to those meeting criteria for PTSD (*M* = 13.86, *SD* = 3.59) or neither (*M* = 12.40, *SD* = 5.26).

Another between-subjects MANOVA was used to assess differences among PTSD, DSO and TALE scores. This was also significant (*F*_6,264_ = 27.16, *p* < 0.001, Wilk’s Λ = 0.38). Follow-up univariate ANOVAs showed significant differences between PTSD (*F*_2_ = 72.80, *p* < 0.001, η_p_^2^ = 0.52), DSO (*F*_2_ = 26.89, *p* < 0.001, η_p_^2^ = 0.29) and TALE scores (*F*_2_ = 14.95, *p* < 0.001, η_p_^2^ = 0.18). *Post-hoc* Tukey’s tests were used in an attempt to replicate prior findings of PTSD symptom severity in people with cPTSD. PTSD scores were significantly higher among those meeting criteria for PTSD (*M* = 15.93, *SD* = 4.42) and cPTSD (*M* = 18.66, *SD* = 3.98) than those who did not meet criteria (*M* = 8.09, *SD* = 5.32). DSO scores were significantly higher among those meeting criteria for cPTSD (*M* = 18.98, *SD* = 3.60) than those meeting criteria for PTSD (*M* = 9.80, *SD* = 4.90) or neither (*M* = 11.90, *SD* = 7.06). Lastly, TALE scores were significantly higher among those meeting criteria for cPTSD (*M* = 11.61, *SD* = 2.93) than those meeting PTSD criteria (*M* = 8.93, *SD* = 3.73) or neither (*M* = 8.39, *SD* = 3.57).

### Hierarchical Regressions

Bivariate Pearson correlations are presented in Table 1. These did not indicate a need for further regressions on excitatory subscales, as no significant relationships were observed with predictor variables. Hierarchical regressions were then used to assess whether DSO scores predicted positive, negative, cognitive and affective PANSS scores, and whether these associations survived the addition of PTSD scores as a covariate (see Table 2 for coefficients). As neither PTSD nor DSOs were significant predictors of negative or cognitive subscale scores, exploratory mediation analyses were only assessed for positive and affective subscales.

**TABLE 2:** Standardized coefficients of hierarchical regressions predicting PANSS scores.

Predictor	Positive	Negative	Cognitive	Affective
**Step 1**				
DSOs	0.383**	0.284*	–0.154	0.606**
*F*	23.737	11.501	3.138	76.773
*R* ^2^	0.212	0.086	0.016	0.363
**Step 2**				
DSOs	0.178	0.184	–0.079	0.436**
PTSD	0.344**	0.171	–0.125	0.295**
Δ*F*	13.533	2.761	1.349	13.267
Δ*R^2^*	0.077	0.019	0.010	0.058

DSOs, Disturbance of self organization; PTSD, Post-traumatic stress disorder. *p < 00.01; **p < 00.001.

### Mediation Analyses

#### Positive Symptoms

**FIGURE 2:**
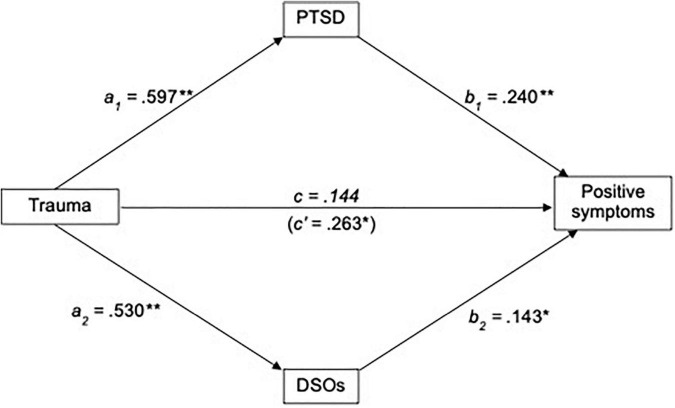
Standardized regression coefficients for the relationship between trauma and positive symptoms, mediated by PTSD and DSOs, including dataset as a covariate. **p < 0.005; *p < 0.05. PTSD, Post-traumatic stress disorder; DSOs, Disturbances of self-organization.

Exploratory parallel mediation analyses were conducted via the SPSS PROCESS macro (model 4; Hayes, 2018) to assess whether PTSD and/or DSOs mediate the relationship between TALE and positive PANSS scores, including dataset as a covariate. TALE scores significantly predicted PTSD (*b* = 0.597, *t*_137_ = 3.989, *p* < 0.001, 95% CI [0.349, 0.845]) and DSOs (*b* = 0.530, *t*_137_ = 3.648, *p* < 0.001, 95% CI [0.290, 0.771]). Dataset also significantly predicted both PTSD (*b* = −3.463, *t*_137_ = −3.097, *p* = 0.002, 95% CI [−5.315, −1.611]) and DSOs (*b* = −4.008, *t*_137_ = −3.689, *p* < 0.001, 95% CI [−5.807, −2.209]). When controlling for PTSD, DSOs and dataset, TALE scores no longer predicted positive PANSS scores (*b* = 0.144, *t*_135_ = 1.338, *p* = 0.183, 95% CI [−0.034, 0.321]). Both PTSD (*b* = 0.240, *t*_135_ = 3.650, *p* < 0.001, 95% CI [0.131, 0.349]) and DSO scores (*b* = 0.143, *t*_135_ = 2.118, *p* = 0.036, 95% CI [0.031, 0.255]) significantly mediated the relationship between TALE scores and positive PANSS scores. Dataset significantly predicted positive PANSS scores (*b* = 1.770, *t*_135_ = 2.235, *p* = 0.027, 95% CI [0.458, 3.082]). Regression statistics are presented in Table 3, and results displayed graphically in Figure 2.

**TABLE 3:** Regression statistics for the mediation pathways predicting psychotic symptoms.

	Positive symptoms			Affective symptoms		
Path	*R* ^2^	*F*	*P*	*R* ^2^	*F*	*p*
*a*_1_	0.201	17.274	<0.001	0.177	27.456	<0.001
*b*_1_	0.211	18.351	<0.001	0.139	20.596	<0.001
*c’*	0.063	10.496	0.001	0.097	15.505	<0.001
*c*	0.257	11.687	<0.001	0.224	12.125	<0.001

*a1 = TALE → PTSD, a2 = TALE → DSO, c’ = TALE → PANSS-Positive, c = TALE → PTSD + DSO → PANSS-Positive. Positive symptom coefficients include dataset as a covariate.*

#### Affective Symptoms

**FIGURE 3:**
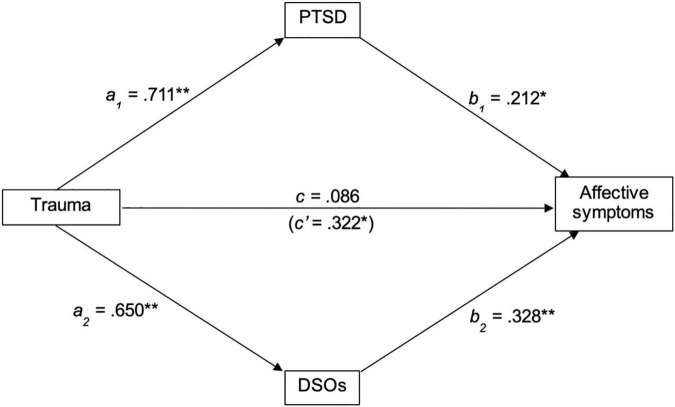
Standardized regression coefficients for the relationship between trauma and affective symptoms, mediated by PTSD and DSOs. **p < 0.005; *p < 0.05. PTSD, Post-traumatic stress disorder, DSOs, Disturbances of self-organization.

A similar mediation analysis was conducted to assess whether PTSD and DSOs mediate the relationship between TALE and affective PANSS scores. TALE scores significantly predicted PTSD (*b* = 0.711, *t*_132_ = 3.998, *p* < 0.001, 95% CI [0.464, 0.958]) and DSOs (*b* = 0.649, *t*_132_ = 4.375, *p* < 0.001, 95% CI [0.403, 0.895]). When controlling for PTSD and DSOs, TALE scores no longer predicted positive PANSS scores (*b* = 0.086, *t*_130_ = 0.840, *p* = 0.403, 95% CI [−0.084, 0.257). PTSD scores significantly mediated the relationship between TALE scores and positive PANSS scores (*b* = 0.212, *t*_130_ = 3.342, *p* = 0.001, 95% CI [0.107, 0.317]), as did DSO scores (*b* = 0.328, *t*_130_ = 5.139, *p* < 0.001, 95% CI [0.222, 0.434]). Regression statistics are presented in Table 3, and results displayed graphically in Figure 3.

## Discussion

To the authors’ knowledge, this is the first study to investigate the frequency and correlates of cPTSD in a trauma-exposed sample of people with psychosis. A higher proportion of the sample met criteria for cPTSD than PTSD. The results suggest that participants meeting criteria for cPTSD presented with significantly higher positive, negative and affective symptoms than those who did not meet criteria for a trauma diagnosis. These did not differ from those meeting criteria for PTSD, aside from affective symptoms, which were significantly higher in the cPTSD group.

The frequency of cPTSD in our sample may suggest that PTSD symptoms in psychosis occur in the context of cPTSD. In this sense, our mediation models may hint at the role of cPTSD in the trauma-psychosis pathway. Consistent with hypotheses, PTSD and DSO symptoms mediated the relationship between trauma and positive symptoms. Of note, the DSO path became significant following the inclusion of dataset as a covariate. The mediation analyses were rerun in each dataset to tentatively investigate the validity of this effect; the coefficients of the DSO path closely resembled that of the adjusted, pooled analysis reported above, though these did not reach statistical significance in either dataset. This pattern of results suggests the samples of each parent dataset were inadequately powered to test these multivariate models individually. This preserves the validity of our results, but clearly requires further replication in larger samples. Also as hypothesized, both PTSD and DSOs were found to mediate the relationship between trauma and affective symptoms. However, significant relationships between excitative symptoms, PTSD and DSOs were not observed, and neither PTSD nor DSOs significantly predicted negative or cognitive symptoms, contraindicating further regression and mediation analyses.

Our main aim of assessing how common cPTSD is relative to PTSD in a trauma-exposed sample of people with psychosis was met. The considerable proportion of participants meeting criteria for cPTSD is consistent with prior research demonstrating the prevalence of difficulties consistent with DSOs among those with psychosis (Beck et al., 2019; Alameda et al., 2020; Sideli et al., 2020). The finding that cPTSD may be more common than PTSD is consistent with other clinical samples (e.g., Karatzias et al., 2017; Griffiths and McLeod, 2020; DeTore et al., 2021). This may be unsurprising, considering the prevalence of complex interpersonal trauma in this population, thought to underpin cPTSD (see (Cloitre et al., 2013). We did not replicate prior findings of increased PTSD symptom severity in people meeting cPTSD criteria (Wolf et al., 2015; Murphy et al., 2016), though our descriptive statistics between groups hint at these findings in larger samples of people with psychosis. These results demonstrate the importance of trauma-informed approaches to assessment and treatment of those with psychosis, owing to the potentially high prevalence of cPTSD that larger epidemiological studies could confirm. Again, this could contextualize the reliability of PTSD mediating the trauma-psychosis relationship in terms of cPTSD, with specific symptoms thereof predicting specific psychotic phenomena. This is consistent with prior findings (Bloomfield et al., 2021), but requires further empirical investigation.

The second aim of this study exploring the relative contribution of cPTSD via PTSD and DSOs in explaining the relationship between trauma and psychotic symptoms was also met. The finding that PTSD symptoms mediate the relationship between trauma and positive symptoms is consistent with prior systematic reviews (Williams et al., 2018; Alameda et al., 2020; Sideli et al., 2020) and meta-analyses (Bloomfield et al., 2021). Indeed, it may be that re-experiencing symptoms of PTSD underpin positive symptoms in certain psychosis subgroups (Hardy, 2017). The finding that DSOs also mediate this is not surprising, owing to the theoretical consistency between these and related constructs previously shown to predict hallucinations and delusions (Williams et al., 2018; Alameda et al., 2020). Notably, however, the effect size of the PTSD path is almost double that of the DSO path, suggesting the way in which cPTSD may precipitate or maintain positive symptoms may primarily occur via PTSD symptoms, rather than DSOs. This finding could be explained by the sensory phenomenology of re-experiencing symptoms, compared to the psychological phenomenology of DSOs in cognitive and emotional patterns (Dorrepaal et al., 2012), leaving core PTSD symptoms open to interpretation as anomalous experiences (Morrison et al., 2003). That said, re-experiencing symptoms have been shown not to correlate with positive PANSS scores (Hamner et al., 1999). Indeed, recent network analyses have demonstrated that trauma-related beliefs and hypervigilance may be more closely related to positive symptoms of psychosis than re-experiencing symptoms (Hardy et al., 2020). Therefore, it may be that the complexity of trauma histories among those with cPTSD lead to trauma beliefs dissimilar to those with PTSD that predict positive symptoms. However, qualitative (Stadtmann et al., 2018) and quantitative (Karatzias et al., 2018) research suggests that trauma-related beliefs among those with cPTSD are similar to those with PTSD. The present study therefore requires replication to assess the reliability of these findings, to further delineate the mechanism by which DSOs may impact psychotic symptoms.

Our findings suggest that cPTSD may underpin the affective difficulties among people with psychosis. This would be unsurprising, considering cPTSD is more strongly correlated with anxiety and depressive disorders than PTSD (Hyland et al., 2018; Karatzias et al., 2019). Affective difficulties (i.e., anxiety and depressive disorders) are highly comorbid with psychosis (Buckley et al., 2009). Prospective studies suggest anxiety and depression mediate the relationship between childhood victimization and adolescent psychotic-like experiences (Fisher et al., 2013), consistent with the affective pathway to psychosis (Myin-Germeys and van Os, 2007). Affective difficulties may also maintain psychoses, posing a higher risk of maladaptive appraisals and behavioral responses to psychotic experiences that perpetuate the experience (Kuipers et al., 2006). Together with our findings, this literature could imply an aetiological or maintaining role of cPTSD in the affective pathway to psychosis. This requires exploration in longitudinal studies, which could indicate the adjustment of trauma-focused cognitive behavior therapy for psychosis to account for cPTSD symptoms. Considering our model clustered DSOs together, such research may investigate symptom-specific relationships between cPTSD and psychosis to uncover finer mechanisms for therapeutic targets. These were not explored here following considerations of statistical power.

The small correlation between negative symptoms of psychosis and all trauma-related variables is consistent with the literature. Meta-analytic evidence has found no relationship between childhood trauma and negative symptoms (Bailey et al., 2018), and that TF-CBT may not lead to significant improvements in this domain (Brand et al., 2018). Whilst reliable, these findings seem at odds with the consistency between negative and post-traumatic sequelae [e.g., interpersonal difficulties and social withdrawal, (Griffiths and McLeod, 2020)], as well as recent network analyses demonstrating paths between specific adverse childhood experiences and negative symptoms (Isvoranu et al., 2017). Negative symptoms may be divided conceptually into experiential and expressive subgroups (Brian Kirkpatrick, 2014); it may be that the PANSS score does not reflect this complexity that is captured by, for instance, the Scale for the Assessment of Negative Symptoms (Andreasen, 1989). Similar arguments may be applied to the measurement of positive symptoms in this study, considering experiences such as hallucinations and delusions were collapsed into a single score, despite research demonstrating specific trauma pathways to each (Hardy, 2017; Bloomfield et al., 2021). Future studies may therefore adopt a symptom-specific approach to assess whether and how trauma and post-traumatic sequelae may predict specific psychotic symptoms. Prior studies have also shown no relationship between cognitive or excitative symptoms and PTSD (Lysaker and LaRocco, 2008; DeTore et al., 2021); our findings are consistent with these. It is outlined above that high cognitive/excitative symptoms may reflect a subgroup of individuals experiencing psychosis via a neurodevelopmental pathway. Whilst atypical neurodevelopment may pose a risk for childhood victimization, this may not necessarily incur post-traumatic symptomatology (Liu et al., 2021). This further demonstrates the importance of assessing for trauma-related difficulties in psychosis, to inform whether said difficulties are incorporated into formulations and treatment plans.

A number of methodological limitations should be noted. For instance, the relatively small number of participants meeting criteria for PTSD may have reduced statistical power – G*Power analyses indicate an achieved power of 0.4 in the comparison of positive symptoms between trauma groups, far below the acceptable 0.8 (Cohen, 1988). Our finding that those meeting criteria for neither PTSD nor cPTSD significantly differed in psychotic symptoms to those meeting cPTSD criteria may indicate a stepwise increase in psychotic symptom severity along the spectrum of trauma-related diagnoses. Future research in larger samples may better assess this, to delineate the clinical utility of diagnosing cPTSD separately from PTSD among those with psychosis.

The unidimensional measurement of emotional dysregulation on the ITQ may not represent the complexity of affective difficulties among those with cPTSD. The ITQ currently includes two items – one assessing emotional hyperactivation and the other hypoactivation – that are summed to derive a single emotional dysregulation score, as per ICD-11 guidance (Maercker et al., 2013). Prior studies suggest that a bifactor structure of this DSO reflecting these dimensions - and measured by more items - may provide a better fit (Ben-Ezra et al., 2018; Murphy et al., 2020). This would align with research (e.g., DeTore et al., 2021) demonstrating the specificity of certain traumas with specific affective regulatory difficulties. Further, as a self-report measure, diagnostic categories assigned by the ITQ may not be as valid as, for instance, a structured clinical interview. The ITQ is the only validated psychometric measure of cPTSD, though Lechner-Meichsner and Steil (2021) propose updates to the Clinician-Administered PTSD Scale to diagnose cPTSD. Perhaps these limitations may be considered in ongoing development of the ITQ.

Our models could not be adjusted for potential clinical confounds. Substance misuse is common among trauma survivors (Ayres, 2020) and has been shown to predict psychosis (Arseneault et al., 2002), but was unfortunately not measured by the parent datasets. This could arguably act as a covariate in our model, though this is unclear based on prior systematic reviews (Williams et al., 2018). Another potential confound is dissociation – an associated, but not core, feature of both PTSD and cPTSD that has been shown to mediate the relationship between trauma and psychosis (Williams et al., 2018; Alameda et al., 2020). Similarly, further exploration of contextual factors (e.g., lack of emotional resources) that may moderate the expression of complex trauma symptoms and their relationships with psychotic outcomes is warranted. Future research controlling for these confounds could elucidate further the relative contribution of cPTSD in explaining the trauma-psychosis relationship.

Owing to the cross-sectional nature of this study, causal interpretations of our model should be very tentative. The possibility of reverse causation from positive and/or affective symptoms to PTSD and DSOs cannot be completely discounted, though previous prospective studies make this unlikely (Pastore et al., 2020). Paradigms focused on finer-grained measurement of cPTSD and psychosis may prove fruitful in future research refining these models. One paradigm - ecological sampling methods (ESM) – may address both questions, enabling a fine-grained assessment of whether DSOs and psychosis interact in the flow of daily life. One study (Kimhy et al., 2020) found emotion regulation predicted psychosis symptoms in daily life, despite no association between retrospective measures of the same variables, demonstrating the utility of ESM in overcoming common methodological limitations (e.g., recall bias).

The sample may constitute a limitation of this study. For one, it is predominantly white. The prevalence of psychosis in minoritised ethnic groups (Qassem et al., 2015) coupled with the trauma of systemic racial discrimination could mean psychotic symptoms and DSOs interact differently in minoritised groups than in their white counterparts. Indeed, systemic racism may decrease self-concept clarity (Kogan et al., 2015), known to be common in people with psychosis (Cicero et al., 2016) building the plausibility of this argument. Future research into this area may explore the generalisability of these findings to black and minority ethnic populations; such research may have implications for sociodevelopmental pathways to psychosis (see (Morgan et al., 2010). Including those with affective psychosis may pose a further limitation of this sample, considering biological sequelae robustly mediate the relationship between trauma and bipolar disorder, as opposed to symptoms of post-traumatic stress (Aas et al., 2016). This heterogeneity may have diluted the effects of cPTSD in this sample. However, diagnoses were not collected using gold-standard tools. They were validated with clinical services and medical notes, though exact confounding effects are therefore difficult to determine. Future studies into the impact of cPTSD in psychosis may choose to focus specifically on non-affective psychosis samples to avoid such effects.

Our findings suggest a potentially nuanced impact of cPTSD in people with psychosis. Rates of cPTSD in our sample suggest that the ‘core’ symptoms of PTSD (i.e., re-experiencing, hyperarousal and avoidance) mostly occur in the context of cPTSD, impacting or maintaining positive symptoms. Both symptom domains of cPTSD (i.e., PTSD and DSOs) may play a role in the maintenance of positive symptoms and affective difficulties (i.e., anxiety and depression) among people with psychosis. Therefore, whilst trauma-focused interventions may be effective at addressing symptoms of trauma and psychosis in people with psychosis (Van Den Berg et al., 2015), a broader range of treatment options may need to be developed to address DSO-related difficulties. For example, dialectic behavioral skills training, demonstrated as most effective when used as an adjunct therapy (Valentine et al., 2015) and more effective for cPTSD than cognitive processing therapy (Bohus et al., 2020). In any case, diagnostic criteria on the ITQ require a functional impact of DSOs in at least one domain, demonstrating an increased treatment need among those with psychosis and comorbid cPTSD. Future research testing the reliability of our findings in larger, more representative samples may therefore have important assessment and treatment implications as trauma-informed care becomes the norm in psychosis services. Such research could employ intense longitudinal designs and adjust for potential confounds to further refine our models.

## Conclusion

This is the first study to the authors’ knowledge investigating the frequency of cPTSD in a trauma-exposed psychosis sample. In accordance with prior research, cPTSD was more common than PTSD in this sample, comprised of both early and chronic psychosis presentations. The functional impairment required to meet cPTSD criteria, as well as the potentially maintaining role cPTSD in positive and affective psychotic symptoms, demands further research into the impact of this comorbidity. This impact may be more nuanced than first thought, with symptom-specific relationships affecting individuals in different ways. Future research may investigate the relationship between symptoms of cPTSD and psychosis at a momentary level, to assess this potentially dynamic interplay between cPTSD and psychosis, as well as the reliability of these results.

## Data Availability Statement

The data analyzed in this study is subject to the following licenses/restrictions: The dataset analyzed in this study is not publicly available to protect the sensitivity and confidentiality of the participants. Requests to access these datasets should be directed to PP, peter.panayi@manchester.ac.uk.

## Ethics Statement

The studies involving human participants were reviewed and approved by National Institute for Health Research, National Health Service. The patients/participants provided their written informed consent to participate in this study.

## Author Contributions

PP, FV, KB, and WS contributed to the conception and design of the study. FV, WS, CC, and RB were involved in collecting the data. PP analyzed the data, and wrote the first draft of the manuscript. FV, KB, WS, and RB contributed to the final draft of the manuscript. All authors contributed to the article and approved its submission.

## Author Disclaimer

The views expressed are those of the authors and not necessarily those of the NIHR or the Department of Health and Social Care.

## Conflict of Interest

The authors declare that the research was conducted in the absence of any commercial or financial relationships that could be construed as a potential conflict of interest.

## Publisher’s Note

All claims expressed in this article are solely those of the authors and do not necessarily represent those of their affiliated organizations, or those of the publisher, the editors and the reviewers. Any product that may be evaluated in this article, or claim that may be made by its manufacturer, is not guaranteed or endorsed by the publisher.
