# Supplementary material for: The Role and Clinical Correlates of Complex Post-traumatic Stress Disorder in People With Psychosis
Source: Front Psychol. 2022 Mar 16;13:791996. doi: 10.3389/fpsyg.2022.791996 (PMC8967251; doi:10.3389/fpsyg.2022.791996)
Supplement: Supplementary file 1 [file Data_Sheet_1.docx]

Supplementary Material

# Supplementary Tables

**Supplementary Table 1.** Breakdown of pentagonal PANSS factor structure by item

| **Positive** | **Negative** | **Cognitive** | **Affective** | **Excitative** |
| --- | --- | --- | --- | --- |
| P1 | N2 | P2 | G2 | P7 |
| G9 | N1 | G11 | G6 | G14 |
| P3 | N4 | N5 | G3 | P4 |
| P6 | N6 | G13 | G4 | G8 |
| P5 | N3 | N7 | G1 |  |
|  | G7 | G5 |  |  |
|  | G16 | G15 |  |  |
|  |  | G10 |  |  |

*^Note: P = Positive, N = negative, G = general psychopathology^*

**Supplementary Table 2.** Independent samples *t*-tests investigating dataset differences

| Subscale | Dataset (*M[SD]*) | | *t* | *df* | *p* |
| --- | --- | --- | --- | --- | --- |
|  | EASE | CC |  |  |  |
| Positive | 15.66(3.56) | 15.22(5.47) | .539^1^ | 141.48 | .56 |
| Negative | 16.43(5.63) | 12.18(3.54) | 4.85^1^ | 74.07 | <.001 |
| Affective | 17.79(4.28) | 12.51(4.74) | 6.56 | 135 | <.001 |
| Cognitive | 12.75(2.82) | 13.31(5.04) | -.73 | 133 | .47 |
| Excitative | 4.39(1.02) | 5.32(2.84) | -2.77^1^ | 112.34 | .007 |
| TALE score | 10.88(3.31) | 8.95(3.67) | 3.20 | 141 | .002 |
| PTSD | 15.98(6.39) | 11.33(6.66) | 4.20 | 140.0 | <.001 |
| DSO | 17.58(4.79) | 12.52(7.12) | 4.96^1^ | 138.82 | <.001 |

*_Note: PANSS = Positive and Negative Syndrome Scale, TALE = Trauma and Life Events Checklist, PTSD = Post-traumatic Stress Disorder, DSO = Disturbances of Self-Organisation._ ^1^_Equal variances not assumed._*

**Supplementary Table 3.** Demographic and clinical characteristics of the sample (N = 144)

| *Age (Mean[SD])* | *Years* | *40.7(13.8)* |
| --- | --- | --- |
| *Gender (%)* | *Male* | *57.6* |
|  | *Female* | *42.4* |
| *Nationality (%)* | *British* | *95.2* |
|  | *Other* | *4.8* |
| *Ethnicity (%)* | *White - Caucasian* | *84* |
|  | *Asian* | *4.9* |
|  | *Black* | *4.9* |
|  | *Mixed heritage* | *5.6* |
|  | *Other* | *.7* |
| *Relationship status (%)* | *Never married* | *69.0* |
|  | *Married or cohabiting* | *19.0* |
|  | *Separated* | *2.1* |
|  | *Divorced or annulled* | *9.9* |
| *Education (%)* | *Primary education* | *21.0* |
|  | *Secondary education* | *30.0* |
|  | *Accessed further/higher education* | *49.0* |
| *Employment (%)* | *Unemployed* | *74.1* |
|  | *Working* | *13.3* |
|  | *Studying* | *3.5* |
|  | *Retired* | *6.3* |
|  | *Other* | *2.8* |
| *Diagnosis (%)* | *Schizophrenia^1^* | *47.2%* |
|  | *Affective psychosis^2^* | *23.6%* |
|  | *Psychosis NOS^3^* | *29.2%* |
| *Antipsychotic medication (%)* | *Yes* | *85.4%* |
|  | *No* | *14.6%* |

*_Note: TALE = Trauma and Life Events Checklist, NOS = Not Otherwise Specified._ ^1^_Including those diagnosed with ‘schizophrenia’, ‘paranoid schizophrenia’ and ‘treatment-resistant schizophrenia’._ ^2^_Including those diagnosed with ‘bipolar affective disorder’, ‘schizoaffective disorder’, or ‘psychotic depression’._ ^3^_Including those diagnosed with ‘psychosis’, ‘first episode psychosis’, ‘non-organic psychosis’ and ‘PTSD with psychotic symptoms’._*

**Supplementary Table 4.** Standardised coefficients and confidence intervals of the meta-analytic mediation model

| *Path* | *b* | *LBCI* | *UBCI* |
| --- | --- | --- | --- |
| a^1^ | 0.62 | 0.51 | 0.73 |
| a^2^ | 0.62 | 0.49 | 0.74 |
| b^1^ | 0.36 | 0.14 | 0.60 |
| b^2^ | 0.19 | -0.05 | 0.43 |
| c | 0.58 | -0.32 | NA |

*_Note:_* *_a1_* _= TALE 🡪 PTSD,_ *_a2_* _= TALE 🡪 DSO,_ *_b1_* _= PTSD 🡪 PANSS-Positive,_ *_b2 =_* _DSO 🡪 PANSS-Positive,_ *_c_* _= TALE 🡪 PTSD + DSO 🡪 PANSS-Positive. LBCI = Lower-bound confidence interval; UBCI = Upper-bound confidence interval._
